# Deletion of *Xist* repeat B disrupts cell cycle and asymmetric cell division through *Usp9x* hyperactivation in mice

**DOI:** 10.1093/nar/gkaf142

**Published:** 2025-03-05

**Authors:** Mingming Liang, Lichao Zhang, Heng Gong, Li Yang, Haijun Wang, Na Song, Liangxue Lai, Wanhua Xie, Zhanjun Li

**Affiliations:** Laboratory of Organ Regeneration and Transplantation of The Ministry of Education, China-Singapore Belt and Road Joint Laboratory on Liver Disease Research, The First Hospital of Jilin University, Changchun 130021, China; CAS Key Laboratory of Regenerative Biology, Guangdong Provincial Key Laboratory of Stem Cell and Regenerative Medicine, Guangzhou Institutes of Biomedicine and Health, Chinese Academy of Sciences, Guangzhou 510530, China; State Key Laboratory for Diagnosis and Treatment of Severe Zoonotic Infectious Diseases, Key Laboratory for Zoonosis Research of the Ministry of Education, Institute of Zoonosis, and College of Veterinary Medicine, Jilin University, Changchun 130062, China; State Key Laboratory for Diagnosis and Treatment of Severe Zoonotic Infectious Diseases, Key Laboratory for Zoonosis Research of the Ministry of Education, Institute of Zoonosis, and College of Veterinary Medicine, Jilin University, Changchun 130062, China; State Key Laboratory for Diagnosis and Treatment of Severe Zoonotic Infectious Diseases, Key Laboratory for Zoonosis Research of the Ministry of Education, Institute of Zoonosis, and College of Veterinary Medicine, Jilin University, Changchun 130062, China; School of Basic Medical Sciences, Xinxiang Medical University, Xinxiang 453000, China; School of Basic Medical Sciences, Xinxiang Medical University, Xinxiang 453000, China; CAS Key Laboratory of Regenerative Biology, Guangdong Provincial Key Laboratory of Stem Cell and Regenerative Medicine, Guangzhou Institutes of Biomedicine and Health, Chinese Academy of Sciences, Guangzhou 510530, China; State Key Laboratory for Diagnosis and Treatment of Severe Zoonotic Infectious Diseases, Key Laboratory for Zoonosis Research of the Ministry of Education, Institute of Zoonosis, and College of Veterinary Medicine, Jilin University, Changchun 130062, China; Center for Medical Epigenetics, School of Basic Medical Sciences, Chongqing Medical University, Chongqing 400016, China; Laboratory of Organ Regeneration and Transplantation of The Ministry of Education, China-Singapore Belt and Road Joint Laboratory on Liver Disease Research, The First Hospital of Jilin University, Changchun 130021, China; College of Animal Science, Jilin University, Changchun 130062, China; Sanya Institute of Swine Resource, Hainan Provincial Research Center of Laboratory Animals, Sanya 572000, China

## Abstract

During X chromosome inactivation (XCI), *Xist* RNA establishes silencing by coating the chromosome in *cis* and binding diverse proteins to promote formation of a heterochromatic domain. However, *Xist* repeat B role beyond initiation of XCI remains unclear. Here, we find that loss of *Xist* repeat B in female mice allows survival and leads to a small body size persisting throughout life. Epigenetic and transcriptomic analyses reveal low levels of H3K27me3 and H2AK119ub occupancy on the X chromosome, except in certain CpG island regions, and partial reactivation of X-linked genes on the inactive X across multiple tissues. Notably, overdosage of *Usp9x* promotes centrosome amplification and chromosome instability. We further demonstrate that *Usp9x* overdosage alters asymmetric cell division, thereby affecting the process of cell differentiation. Thus, *Xist* repeat B is necessary for gene-specific silencing during XCI maintenance and impacts cell proliferation and differentiation during development. This provides insights into repeat B importance in maintaining XCI.

## Graphical Abstract

**Graphical Abstract:**
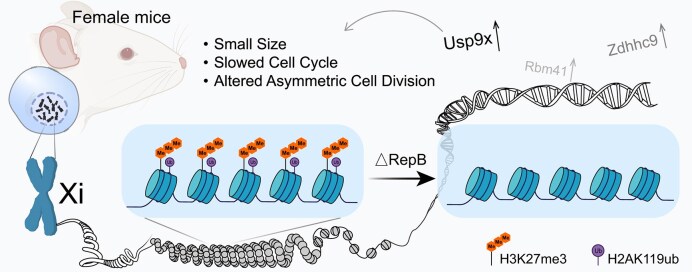

## Introduction

X chromosome inactivation (XCI) is a dosage compensation phenomenon that occurs in mammalian females to equalize the dosage of X-linked genes through transcriptional silencing of one of the two X chromosomes [1]. XCI occurs in two waves during female mouse development. A first wave of imprinted paternal X (Xp) inactivation occurs in preimplantation embryos. As development proceeds, the silent Xp is maintained in extra-embryonic tissues. However, the Xp is briefly reactivated in the inner cell mass that form the epiblast. In this second wave of silencing, XCI occurs randomly on either the Xp or maternal X (Xm) [2, 3]. During XCI, the long noncoding RNA *Xist* is transcribed exclusively from the future inactive X (Xi) and coats it in *cis*, triggering gene silencing and a cascade of chromatin changes, including eviction of activating factors and recruitment of silencing factors, such as Polycomb repressive complexes 1 and 2 (PRC1, PRC2) [4–12]. Once established, the Xi is remarkably stable due to the retention of repressive heterochromatin.

With the establishment of the transcriptionally silent state, cells transition into the XCI maintenance phase in which the same X chromosome remains Xi through subsequent cell divisions. In the maintenance phase, *Xist* remains highly expressed and anchored to the Xi. Proper maintenance of the silent state of the Xi is essential for female health, as defects in XCI maintenance have been linked to a range of human cancers and developmental disorders [13–21].

Regarding the establishment of repressive heterochromatin, two repeat sequences within *Xist* RNA, known as repeats A and B, have been associated with it. Repeat A initiates gene silencing and Polycomb recruitment, whereas repeat B stabilizes them [4, 5, 22–24]. The significance of repeat A is widely accepted [25, 26]. The reports on repeat B region have argued for varying degrees of silencing defects upon its deletion [4, 5, 27, 28]. Some reports show that loss of Polycomb recruitment has only a minor effect on Xi silencing [29, 30], whereas others show a significant effect [4, 5, 12, 28, 31]. These divergent findings have been challenging to reconcile and necessitate further investigation to elucidate the functional of repeat B in Xi gene silencing and Polycomb recruitment during the maintenance phase.

*Xist* is continuously expressed in somatic cells throughout the lifetime of the female to maintain the silent state of the Xi. However, the impact of repeat B loss on female development, X-linked gene expression, and the maintenance of Xi have not yet been addressed. Here, we carry out a systematic deletional analysis of endogenous *Xist* repeat B in mice. We show that deleting *Xist* repeat B in mice is compatible with survival but results in persistent growth retardation. Loss of repeat B caused significant overall reduction of H3K27me3 and H2AK119ub on Xi relative to wild type (WT). Nevertheless, a significant proportion of CpG islands (CGIs) retained enrichment of H3K27me3 or H2AK119ub, respectively. We provide evidence that repeat B loss leads to transcriptional reactivation on the Xi during XCI maintenance. Our mechanistic studies further reveal that reactivation and increased dosage of *Usp9x* leads to cell cycle slowing and asymmetric cell division (ACD) alteration in mice. Collectively, our results indicate that repeat B exerts important roles in controlling repression of transcription on the Xi during XCI maintenance. Our findings on the role of *Xist* repeat B in regulation of expression of X-linked genes will provide valuable insights for developing treatment strategies for X-linked diseases by manipulating the expression of specific X-linked genes on the Xi.

## Materials and methods

### Animals care and use

The Institutional Animal Care and Use Committee of Jilin University approved all animal experiments (IACUC number: SY202302002). All animal procedures were performed per the ethical guidelines of the Jilin University Laboratory Animal Center. Institute of Cancer Research (ICR) female mice aged 8 weeks were purchased from Liaoning Changsheng biotechnology co., Ltd. Mice were housed in a 14-h light/10-h dark cycle at 20–23°C and 40%–50% humidity.

### *Xist* repeat B deletion mice

*Xist* repeat B deletion were generated by CRISPR/Cas9 using a pair of single-guide RNAs (sgRNAs) flanking the target region. Two sgRNAs were designed according to previous described and cloned into the *BbsI*-linearized pUC57-T7-sgRNA vector. Then, sgRNAs were amplified by polymerase chain reaction (PCR) using the primer pair listed in Supplementary Table S1 and *in vitro* transcription using the MAXIscript T7 kit (Invitrogen) and purified with a miRNeasy mini kit (QIAGEN) according to the manufacturer’s instructions. To produce SpCas9 messenger RNA (mRNA), the PCS2 + Cas9 (Plasmid #122948) plasmid was linearized with *NotI* restriction digestion and used as a template to *in vitro* transcribe mRNAs using mMESSAGE mMACHINE SP6 Transcription Kit (Invitrogen) and then Cas9 mRNAs were purified with a miRNeasy mini kit (QIAGEN). All sgRNA sequences are listed in Supplementary Table S1. A mixture of Cas9 mRNA (100 ng/μl) and sgRNA (9 ng/μl) was co-injected into the cytoplasm of pronuclear stage zygotes. Finally, 25–35 injected zygotes were transferred into the oviduct of recipient mice. For mice genotyping, toes were collected and genomic DNA was extracted for PCR. All the primers used in this study are listed in Supplementary Table S1.

### Cell culture

Primary mouse embryonic fibroblasts (MEFs) and HEK293T cell lines were cultured in high-glucose Dulbecco’s modified Eagle’s medium (DMEM, Gibco) supplemented with 10% Fetal bovine serum (FBS, Gibco), 1% penicillin–streptomycin (Gibco), and 1% nonessential amino acids (Gibco) at 37°C, 5% CO_2_.

### Statistical analysis of weight and survival

To analyze survival, we conducted regular daily monitoring of the mice. The survival data are from 20 KO mice and 20 control mice. Body weight was recorded weekly. All data are expressed as mean ± standard deviation (SD) from at least three determinations in all experiments. The data were analyzed by Student’s unpaired t-test using GraphPad Prism software. *P* < 0.05 indicated statistical significance (****P < 0.0001).

### Hematoxylin and eosin staining

Hematoxylin and eosin (H&E) staining was performed according to our published protocols [32]. Briefly, the tissues from mice were fixed in 4% paraformaldehyde for 48h, embedded in paraffin wax, and then sectioned for slides. Then, slides were stained with H&E, and viewed under a Nikon ts100 microscope.

### ChIP-seq

ChIP seq was performed as previously described [33]. In brief, primary MEFs were cross-linked in phosphate-buffered saline (PBS) with 1% formaldehyde at room temperature for 10 min and quenched formaldehyde with 125 mM glycine for 5 min. Cross-linked cells were washed twice with cold PBS. Proceed to the sonication step, and Chromatin was fragmented by sonication using a Bioruptor Pico (Diagenode) for 25 cycles with 30 s on and 30 s off. For each precipitation reaction, fragmented chromatin was incubated overnight at 4°C with antibody against H3K27me3 (Abcam 6002) and H2AK119ub (Abcam ab193203). Immune complexes were further pulled down with protein A-Sepharose at 4°C for 1 h and immunoprecipitated DNA was purified by phenol: chloroform extraction and ethanol precipitation in the presence of linear acrylamide. Input DNA (10% of the amount utilized for immunoprecipitation) was prepared from chromatin extracts and used for the normalization of ChIP samples.

### ChIP-seq analysis

ChIP-seq data were mapped to the mouse reference genome (UCSC mm10) using bowtie2 [34]. Significantly enriched regions were next identified by performing MACS2 analysis [35]. Each processed bam file was normalized to reads per kilobase per million using the BamCoverage tool in DeepTools and visualized using the Integrative Genomics Viewer [36]. The table containing mouse transcriptional start sites (TSS) and transcription end site (TES) was obtained from the UCSC table browser.

### Immunofluorescence/RNA FISH

Immunofluorescence/RNA FISH (RNA fluorescence in situ hybridization) was performed as previously described [37]. Briefly, cells grown on glass coverslips were rinsed in PBS, fixed in 4% paraformaldehyde, and permeabilized in PBS/0.5% Triton-X 100 for 10 min at room temperature. After three rapid washes in PBS, samples were blocked for 30 min with 1% bovine serum albumin in PBS. Primary antibodies were added and allowed to incubate at room temperature for 1 h. Cells were washed three times with PBS at room temperature for 5 min each. After incubating with secondary antibody for 30 min at room temperature, cells were washed again with PBS at room temperature for 5 min each. Afterward, cells were postfixed with 4% paraformaldehyde for 10 min at RT (Room Temperature) and rinsed three times in PBS and twice in 2 × Saline-Sodium Citrate (SSC) buffer. Excess of 2 × SSC was removed, and cells were hybridized with *Xist* FISH probe set (Stellaris^®^ FISH Probes, Mouse *Xist* with Quasar^®^ 570 Dye). After the RNA FISH procedure, nuclei were stained with DAPI (4′,6-diamidino-2-phenylindole, Sigma–Aldrich), diluted 1:5000 in 2 × SSC for 5 min at RT. Cells were observed with Olympus FV3000 confocal laser scanning microscope.

### Quantitative image analysis

All image analysis were performed using Fiji [38].

### RNA-seq and analysis

Total RNA was extracted from primary MEFs. Stranded mRNA libraries were constructed by Genewiz and Novogene, and loaded on an Illumina HiSeq/Illumina Novaseq/MGI2000 instrument for sequencing using a 2 × 150 paired-end configuration according to manufacturer’s instructions. Fastq passes filter data were converted to high quality, clean data using Cutadapt. Using the program Hisat2, data were aligned to the reference genome. The DESeq2 Bioconductor package was utilized for the analysis of differential expression. Data-driven prior distributions are incorporated into the estimations of dispersion and logarithmic fold changes, and the Padj of the genes was adjusted to 0.05 to identify those that were differentially expressed.

### Quantitative real-time PCR analysis

Total RNA from tails were isolated using TRIzol reagent (Invitrogen, 15596026) according to the manufacturer’s instructions, and complementary DNA was synthesized from 2000 ng total RNA using PrimeScript RT Master Mix (Takara RR036A). Quantitative real-time PCR (qPCR) was performed in triplicate using standard SYBR green reagents (Biomed MT521) and protocols on a QuantStudio 3 Real-Time PCR system (Applied Biosystems). The target mRNA expression was quantified using the ΔΔCt method and normalized to GAPDH (Glyceraldehyde 3-phosphate dehydrogenase) expression [39]. All primers were designed using IDT (https://sg.idtdna.com/PrimerQuest/Home/Index) and synthesized by Sangon Biotech (Shanghai) Co., Ltd. Primer sequences are listed in Supplementary Table S1.

### Western blot

Western blot was performed as described previously [40]. Briefly, whole-tissue extracts were prepared with RIPA buffer (Radio Immunoprecipitation Assay Lysis buffer) and PMSF (Phenylmethanesulfonyl fluoride) on ice for 30 min. The samples were vortexed briefly (10 min), and then subjected to western blot analyses with the antibody (Anti-CRISPR-Cas9 antibody, Abcam ab204448; USP9X antibody, Proteintech 55054-1-AP; Alpha Tubulin antibody, Proteintech 11224-1-AP; Cep131 Antibody, Affinity Biosciences DF3692; GPSM1 Antibody, Novus NBP3-04743; Beta Actin antibody, Proteintech 20536-1-AP). The images were captured on the Tanon 5200 Imaging System (Tanon).

### Short hairpin RNA knock-down

To knock down *Usp9x*, the short hairpin RNA (shRNA) sequences (listed in Supplementary Table S2) were obtained from Sigma–Aldrich. Briefly, the shRNA is cloned into the pLKO.1 vector. The vectors were co-transfected with psPAX2 (Addgene, #12260) and pMD2.G (Addgene, #12259) into HEK293T cells using Lipofectamine 3000 transfection reagent. Supernatants were collected 48 and 72 h post-transfection. To generate *Usp9x* knock-down cell lines, MEFs were infected with lentivirus for 24 h. Cells were then cultured in selection medium containing 1 μg/ml puromycin for two weeks to obtain stable *Usp9x* knock-down cell lines.

### Immunofluorescence staining

Tissues were collected from RepB^−/−^ and WT mice. The tissues were fixed in 4% paraformaldehyde at 4°C, dehydrated in increasing concentrations of ethanol (70% for 6 h, 80% for 1 h, 96% for 1 h, and 100% for 3 h), cleared in xylene and embedded in paraffin for the histological examination. The 5-μm sections were cut for antibody staining. The stained sections were imaged with a Nikon TS100 microscope.

### Cell proliferation assay

We performed cell proliferation assay using Cell Counting Kit-8 (APExBIO Technology LLC, USA). Absorbance was measured at 450 nm with a microplate reader (Benchmark Plus Microplate Spectrophotometer; Bio-Rad, Hercules, CA, USA).

### Cell cycle analysis by flow cytometry

Propidium iodide (PI) staining was used for cell cycle analysis. Cells were fixed and permeabilized with 70% ethanol at −20°C for overnight. Fixed cells were washed with PBS twice, resuspended with 20 μg/ml of RNase A in PBS, and stained with a final concentration of 10 μg/ml PI in the dark. Cells were subjected to analysis using a BD FACS canto (Becton Dickinson, Franklin Lakes, NJ, USA) and ModFit LT software (Verity Software House).

### Cell cycle synchronization

Cells were arrested in G1 by double thymidine block [41]. MEFs were cultured in DMEM containing 2.5 mM thymidine overnight, washed with PBS, released in DMEM without thymidine for 9 h, and then incubated in DMEM containing 2.5 mM thymidine overnight. At the end of the second thymidine block, the cells were washed free of thymidine as described above.

### Statistical analysis

Quantitative data were analyzed by GraphPad Prism. Multiple group analyses were performed by analysis of variance. Student’s *t*-test was used to determine a significant difference between two groups. *P*-values of <0.05 were considered statistically significant.

## Results

### *Xist* repeat B deletion does not affect mouse fitness

**Figure 1.**
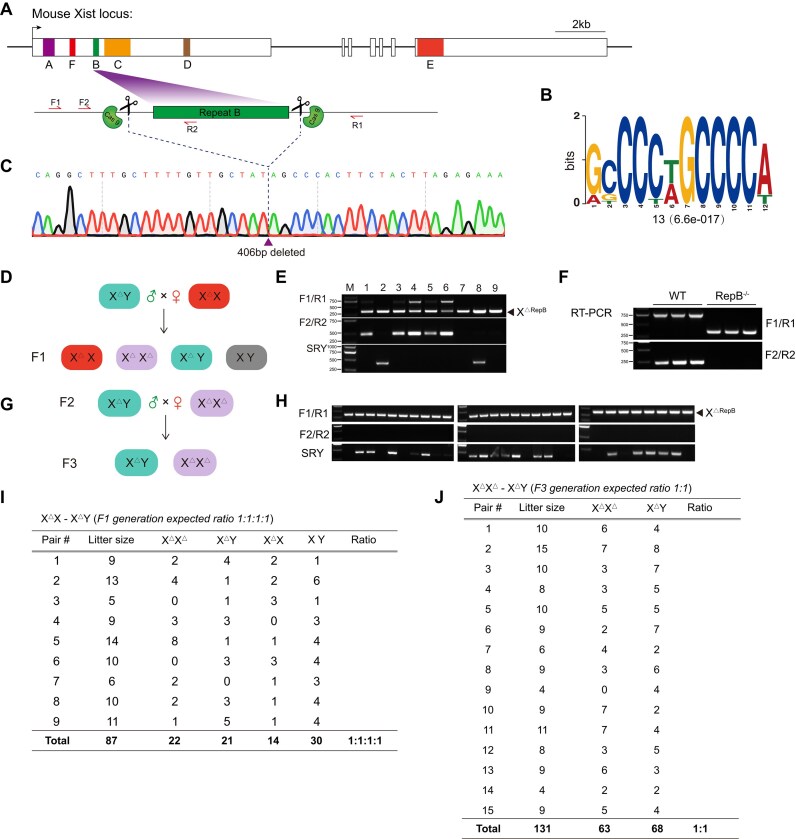
Female mice lacking *Xist* repeat B are viable and produce offspring that follow Mendelian inheritance ratios. (**A**) Map of the *Xist* gene used in this study. A schematic of gene targeting strategy applied to generate *Xist* repeat B knockout mice. Arrows mark the location of the F1/R1 and F2/R2 primer sets (used for detecting the genotype of mice). (**B**) The core motif of *Xist* repeat B is depicted, with the number of sites and the E-value presented below the motif logo. (**C**) The result of Sanger sequencing for the genotype of F0 generation mice. (**D**) Schematic of cross to generate homozygous mice with repeat B deletion. (**E**) Genotype confirmation of F1 generation mice by agarose gel electrophoresis. (**F**) *Xist* gene expression analysis by RT-PCR. (**G**) Mating strategy to validate the reproductive capability of homozygous mice. (**H**) Genotype confirmation of F3 generation mice by agarose gel electrophoresis. (**I**) Table summarizing the litter size and Mendelian ratio of F1 generation mice. (**J**) Table summarizing the litter size and Mendelian ratio of F3 generation mice.

The murine *Xist* gene encompasses six repetitive sequences [42], designated from repeat A through F (Fig. 1A), with repeat B notably characterized by a high cytosine content (Fig. 1B). To elucidate the *in vivo* functionality of repeat B, we employed the CRISPR/Cas9 system to generate mice harboring a targeted deletion of this region (Supplementary Fig. S1A). Genotyping assays were utilized to identify mice with the intended deletion, which was further corroborated by Sanger sequencing in the F0 generation (Fig. 1C). Additionally, to ensure specificity, we examined the top six predicted off-target sites with the highest potential for unintended modifications. The results showed no off-target effects (Supplementary Fig. S1B), and the Cas9 gene was not integrated into the genome of the cloned mice (Supplementary Fig. S1C). To procure homozygous knock-out female mice, we implemented a breeding strategy mating hemizygous males with heterozygous females (Fig. 1D), and the resultant progeny were verified via genotyping (Fig. 1E). Reverse transcriptase-polymerase chain reaction (RT-PCR) revealed that the excision of repeat B within *Xist* did not alter the expression profile of *Xist* (Fig. 1F), suggesting no deleterious impact on the transcription of the gene. To evaluate the reproductive fitness of these female mice, hemizygous males were crossed with homozygous females (Fig. 1G), and subsequent genotyping confirmed that all offspring were indeed homozygous for the deletion (Fig. 1H). To ascertain the normalcy of the offspring distribution, statistical analyses of the F1 and F2 generations were conducted, demonstrating that the frequencies of individuals across both generations adhered to the expected Mendelian inheritance patterns (Fig. 1I and J), thereby validating the lack of detrimental effects on fertility due to the repeat B deletion.

### *Xist* repeat B deficiency results in a dwarf phenotype in mice

**Figure 2.**
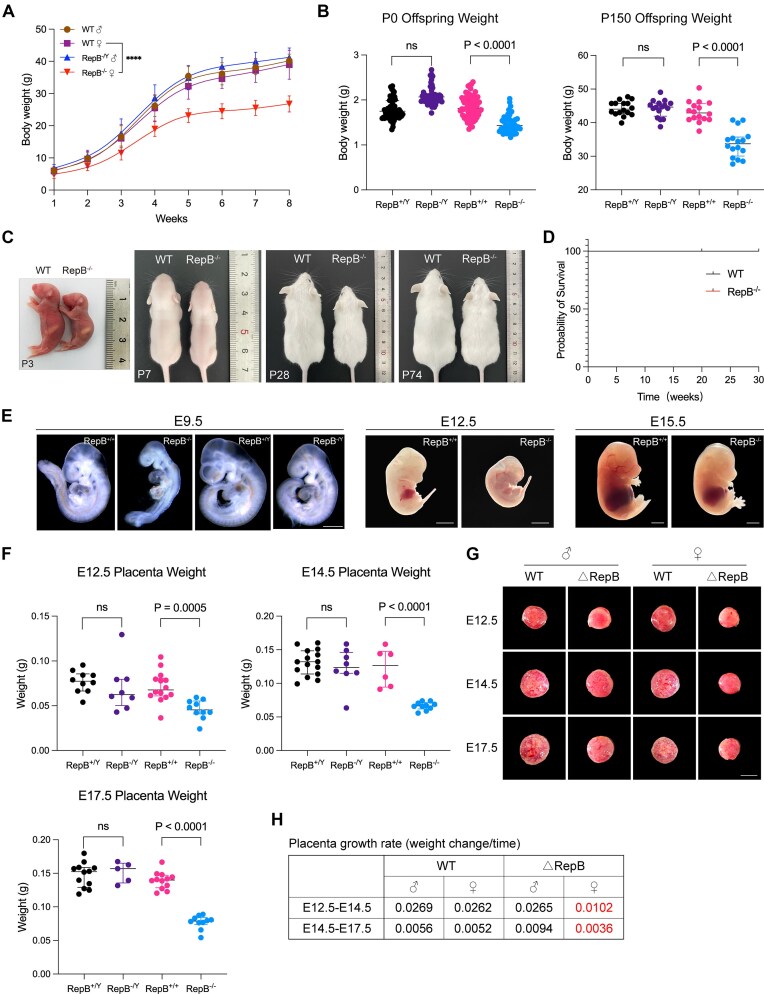
RepB^−/−^ mice exhibit reduced body and placenta sizes. (**A**) Body weight of repeat B knockout and WT mice. Error bars indicate mean ± SD. (**B**) Offspring body weight on the day of birth (P0, *n* = 43–68/genotype) and at postnatal day 150 (P150, *n* = 16/genotype). (**C**) Survival curve for RepB^−/−^ and WT mice. (**D**) Gross appearance of mice of indicated genotypes at various ages. (**E**) Representative morphology of control and KO embryos at E9.5, E12.5, and E15.5. Scale bar, 0.5 mm (E9.5), 2 mm (E12.5 and E15.5). (**F**) Placenta weight of repeat B knockout and WT mice at E12.5, E14.5, and E17.5. (**G**) Gross appearance of repeat B knockout and WT placenta at E12.5, E14.5, and E17.5. Scale bar, 5 mm. (**H**) Table depicting growth rates of placentas in repeat B knockout and WT groups at two gestational time points.

Next, we examined the clinical phenotype arising from the homozygous deletion of repeat B specifically in female and male cohorts. Notably, through continuous monitoring of body weight in mice from birth until eight weeks postnatal, we unveiled a marked reduction in body weight among RepB^−/−^ female mice in contrast to WT females (Fig. 2A and Supplementary Table S3), while males were not significantly affected. Moreover, our statistical assessment of the repercussions of repeat B deficiency on the body weight of mice at birth and 150 days postnatally revealed a significant decrease that was confined to female mice with repeat B deletion (Fig. 2B and Supplementary Table S3). In concordance, the bodily dimensions of RepB^−/−^ females at postnatal days 3, 7, 28, and 74 exhibited pronounced diminution compared to those of WT females (Fig. 2C). Intriguingly, despite these alterations, the absence of repeat B had no discernible impact on female survival rates over a 30-week monitoring period (Fig. 2D). Histopathological inspections further ascertained an absence of significant pathological variations in the heart, liver, spleen, lungs, or kidneys between RepB^−/−^ and WT female mice (Supplementary Fig. S2A). Expanding our investigation, we explored the role of repeat B in early embryonic development, uncovering a conspicuous reduction in the size of female embryos at embryonic stages E9.5, E12.5, and E15.5 in response to repeat B deletion, while no such differences were discerned between RepB^−/Y^ and WT male embryos at E9.5 (Fig. 2E). Furthermore, we evaluated the placental size at E12.5, E14.5, and E17.5 and found that placentas from female mice with repeat B deletion were smaller, whereas male placentas were not significantly affected (Fig. 2F and Supplementary Table S3). Representative images of placental size are shown in Fig. 2G. Moreover, Histopathological inspections indicate that there is no obvious pathological change in placental development (Supplementary Fig. S2B). Additionally, compared with the control group, placental growth rate was reduced in RepB^−/−^ female mice (Fig. 2H). Collectively, these findings indicate that the deletion of *Xist* repeat B specifically influences the development of female mice and their placentas, while having no discernible impact on male mice.

### Deletion of repeat B leads to loss of H3K27me3 and H2AK119ub modifications across the Xi

**Figure 3.**
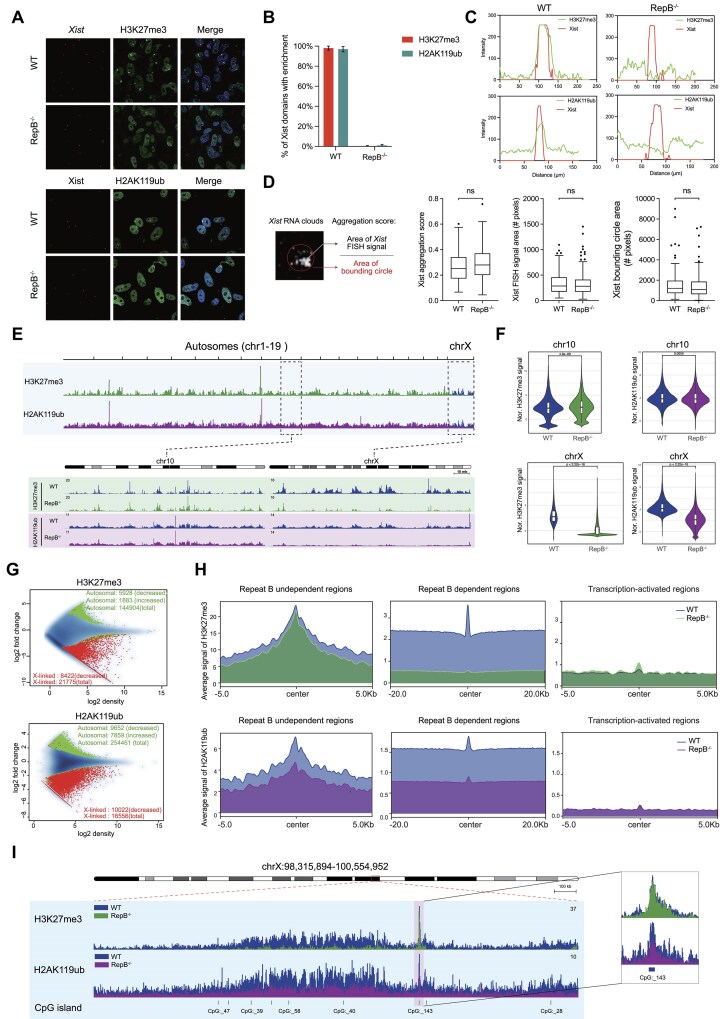
*Xist* repeat B loss leads to epigenetic changes of the Xi. (**A**) H3K27me3/H2AK119ub IF and *Xist* RNA FISH in RepB^−/−^ and WT MEF cells. (**B**) Quantitative analysis of a single FL-*Xist* and RepB^−/−^ cell line based on scoring *Xist* RNA domains for presence or absence of H3K27me3/H2AK119ub. Error bars represent SD for at least three biological replicates with a sample of *n* > 100 for each replicate. (**C**) Green = H3K27me3/H2AK119ub and red = *Xist*. Line intensity profile showing *Xist* and H3K27me3/H2AK119ub intensities across the *Xist* territory in RepB^−/−^ and WT MEF cells. (**D**) Schematic for aggregation score calculation. Box plots showing *Xist* aggregation scores upon depletion of repeat B, *Xist* FISH signal area values and bounding circle area values encompassing the *Xist* signal used to calculate the *Xist* aggregation scores in RepB^−/−^ and WT MEF cells. For box plots (*n* > 180): ns, not significant, two-tailed Kolmogorov–Smirnov test. Horizontal lines denote the median, whiskers indicate 1.5× the interquartile range, dots represent outliers. (**E**) Genome browser tracks showing H3K27me3 (green) and H2AK119ub (purple) accumulation at autosomes and X chromosome in RepB^−/−^ mice. Zoom in H3K27me3 (green) and H2AK119ub (purple) accumulation across the chr10 and chrX regions. (**F**) Violin plots quantifying H3K27me3 and H2AK119ub enrichment over chr10 and chrX regions. (**G**) MA plots showing differences between RepB^−/−^ and WT mice in H3K27me3 or H2AK119ub levels at autosomes and X chromosome. Statistically significant differences are shown in green and red, blue dots and blue density cloud represents all points corresponding to the nonchanging regions. Note that most altered sites show decrease binding on X chromosome in RepB^−/−^ mice. (**H**) ChIP-seq cumulative enrichment deposition centered at peak summit of H3K27me3/H2AK119ub in RepB^−/−^ mice, encompassing regions that are both dependent and independent of repeat B sequences. H3K27me3/H2AK119ub enrichment at transcription-activated regions genome-wide (right panel). (**I**) Genome browser view of H3K27me3/H2AK119ub enrichment at CGIs in RepB^−/−^ mice.

Considering that the *Xist* gene plays a pivotal role in mediating X chromosome inactivation during female embryogenesis through recruitment of PRC1/2 [4,5, 22, 23, 43], we performed immunofluorescence for H3K27me3, H2AK119ub, and RNA-FISH for *Xist* in MEF cells from WT and RepB^−/−^ mice. In nearly all *Xist*-positive WT cells, distinct foci of H3K27me3 and H2AK119ub, hallmarks of inactive X chromatin, clustered near the nuclear periphery. However, in RepB-null cells, immunoFISH analysis revealed complete loss of *Xist* dependent recruitment of H3K27me3 and H2AK119ub deposition (Fig. 3A and B and Supplementary Fig. S3A). The co-localization of *Xist* FISH signals with H3K27me3/H2AK119ub fluorescent signals is lost in RepB^−/−^ cells (Fig. 3C). Additionally, we examined whether the depletion of repeat B affects *Xist* localization. The results of aggregation score calculation revealed that *Xist* remained largely localized (Fig. 3D). These findings indicate that the deletion of repeat B affects the recruitment of H3K27me3 and H2AK119ub, but does not impact *Xist* localization on the Xi.

To further scrutinize these anomalies, we executed ChIP-seq for H3K27me3 and H2AK119ub in WT and RepB^−/−^ mice tail and ear fibroblasts (Supplementary Table S4). Our results illuminated a selective loss in H3K27me3 and H2AK119ub occupancy on the X chromosome in the absence of repeat B, with no discernible effect on autosomes (Fig. 3E and F). Differential occupancy profiling of these modifications, when distinguished between autosomal and X chromosomal contexts, revealed a profound lessening of their abundance at the majority of X-linked sites (Fig. 3G), exhibiting no preferential bias across varied chromatin domains (Supplementary Fig. S4B). In harmony with immunofluorescence outcomes, MEFs from RepB^−/−^ mice showed a widespread loss of H3K27me3 and H2AK119ub signals on the X chromosome (Fig. 3E and Supplementary Fig. S4A), with H3K27me3 being more significantly affected than H2AK119ub. These data indicate that repeat B plays a crucial role in the recruitment of H3K27me3 and H2AK119ub during XCI, consistent with previous findings [4, 5].

We further delineated the regions of X chromatin that are independent and dependent on repeat B. Intriguingly, in the RepB-independent regions, the deletion of repeat B had no impact on H3K27me3 occupancy, as evidenced by unaltered peak heights, but it led to a reduction in H2AK119ub occupancy albeit with a residual presence still evident (Fig. 3H). Conversely, in RepB-dependent regions, the deletion of repeat B resulted in a complete loss of H3K27me3 and a slight residual presence of H2AK119ub, compared to the signal intensity observed in transcription-activated regions (Fig. 3H and Supplementary Fig. S4C). Moreover, these RepB-independent regions were found to be enriched with CGIs (Fig. 3I and Supplementary Fig. S4D). These data imply that silencing of certain CGIs on the X chromosome occurs independently of *Xist* repeat B region.

### Deletion of repeat B specifically upregulated a subset of genes that exhibit a particular chromatin context

**Figure 4.**
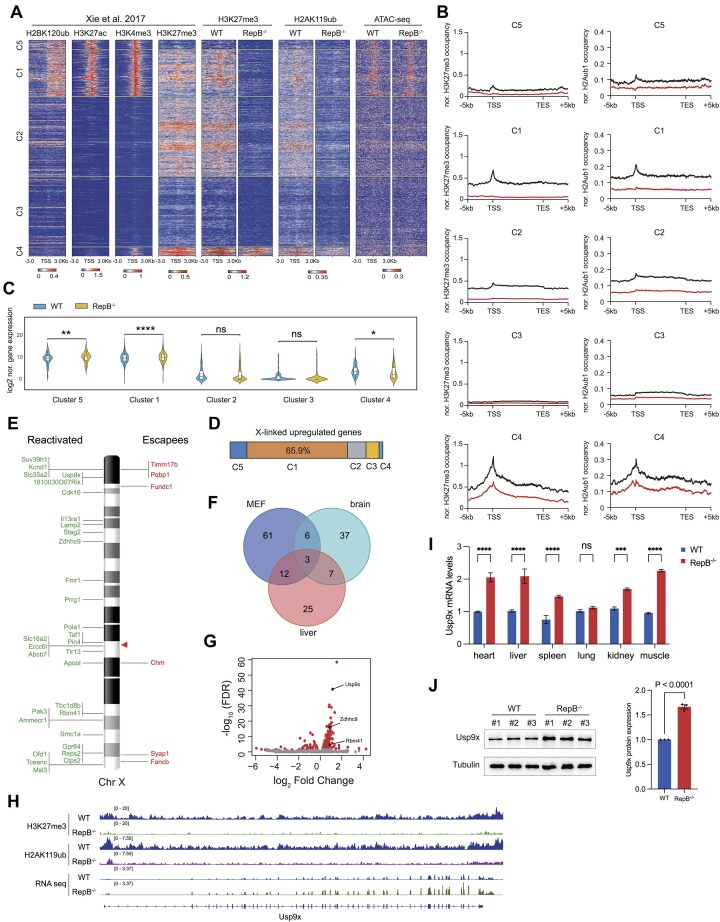
The deletion of *Xist* repeat B leads to reactivation of previously silenced genes on the Xi in RepB^−/−^ mice. (**A**) Heatmaps of H2BK120ub, H3K27ac, H3K4me3, H3K27me3, H2AK119ub, and ATAC-seq levels surrounding the TSS (±3 kb) of all genes in WT and RepB^−/−^ mice. A color scale indicating the relative signal intensity plotted on each heatmap is shown at the bottom. Genes were clustered by class according to the histone modifications occupancy. TSS, transcription start site. Biological replicates ≥2. (**B**) Aggregate plots compare the average H3K27me3 and H2AK119ub ChIP signals over TSS or TES (±5 kb) form clusters C1–C5 in WT and RepB^−/−^ mice. TES, transcription end site. (**C**) Violin plots showing the X-linked gene expression statistics of the different clusters in WT and RepB^−/−^ mice. ns, not significant, *P < 0.05, **P < 0.01, ****P < 0.0001. (**D**) Distribution of upregulated X-linked genes of clusters C1–C5. (**E**) Positioning of escapees (red) and reactivated genes (green) along the X. The red triangle indicates the position of *Xist*. (**F**) Venn diagram shows the number of upregulated X-linked genes shared between the three sampling groups. (**G**) Volcano plot of the fold change of transcripts in RepB^−/−^ MEFs compared to control. The red dots indicate transcripts that showed statistically significant changes. The annotated dots are the common data points shared between the three sampling groups. (**H**) Representative genome tracks show H3K27me3/H2AK119ub ChIP-seq signals and RNA-seq reads across typical *Xist*-dependent XCI reactivated gene *Usp9x* on Xi in RepB^−/−^ MEFs. (**I**) *Usp9x* mRNA levels across various tissues in WT and RepB^−/−^ mice. ns, not significant, ***P < 0.001, ****P < 0.0001. (**J**) *Usp9x* protein expression levels in the tails of WT and RepB^−/−^ mice.

To elucidate the impact of *Xist* repeat B deletion on various X chromatin states, we reanalyzed ChIP-seq datasets for H2BK120ub, H3K27ac, H3K4me3, and H3K27me3 from female mouse MEF cells [33]. Genes on the X chromosome was clustered into five groups according to the presence of these histone marks. Strikingly, our H3K27me3 profile concurred with published data, affirming the stability of X chromatin activity in MEFs (Fig. 4A). Specifically, Cluster 1 (C1) featured both active (H2BK120ub, H3K4me3, H3K27ac) and repressive (H3K27me3) marks; C2, solely H3K27me3; C3, devoid of any marks; C4, a mix of H3K4me3 and H3K27me3; and C5, exclusively active marks (Fig. 4A). Notably, repeat B deletion abolished H3K27me3 and H2AK119ub in C1 and C2 (Fig. 4A and B), while partially reducing these marks in C4, where they remained detectable (Fig. 4A and B). Despite this, repeat B deletion did not alter X chromosome chromatin accessibility (Fig. 4A and Supplementary Fig. S5A and B). We proceeded to investigate X-linked gene expression variations across diverse chromatin landscapes. In line with histone modification changes, C1 and C5 cluster genes showed marked upregulation (Fig. 4C), while C4 cluster genes unexpectedly downregulated in RepB-null tail and ear fibroblasts (Fig. 4C). Notably, around 66% of upregulated X-linked genes clustered in C1 (Fig. 4D and Supplementary Table S5). Additionally, these active genes clustered near the *Xist* locus and the X chromosome termini (Fig. 4E and Supplementary Fig. S5C).

### *Xist* repeat B loss led to *Usp9x* reactivation on Xi across tissues

To identify genes whose repression depends on the *Xist* repeat B element, we conducted RNA sequencing on brain, liver, and fibroblast tissues (Supplementary Table S6). Principal component analysis revealed high consistency among replicates within groups (Supplementary Fig. S5D). Cumulative distribution plots of fold changes from RNA-seq showed a statistically significant rightward shift in X-linked genes compared to autosomal genes across all three tissues (Supplementary Fig. S5E). To further characterize these changes, we binned fold change values in increments of 0.2 and observed a substantial rightward shift in X-linked gene expression relative to autosomal genes (Supplementary Fig. S5F). These results demonstrate that the deletion of repeat B alters the expression profile of X-linked genes.

**Figure 5.**
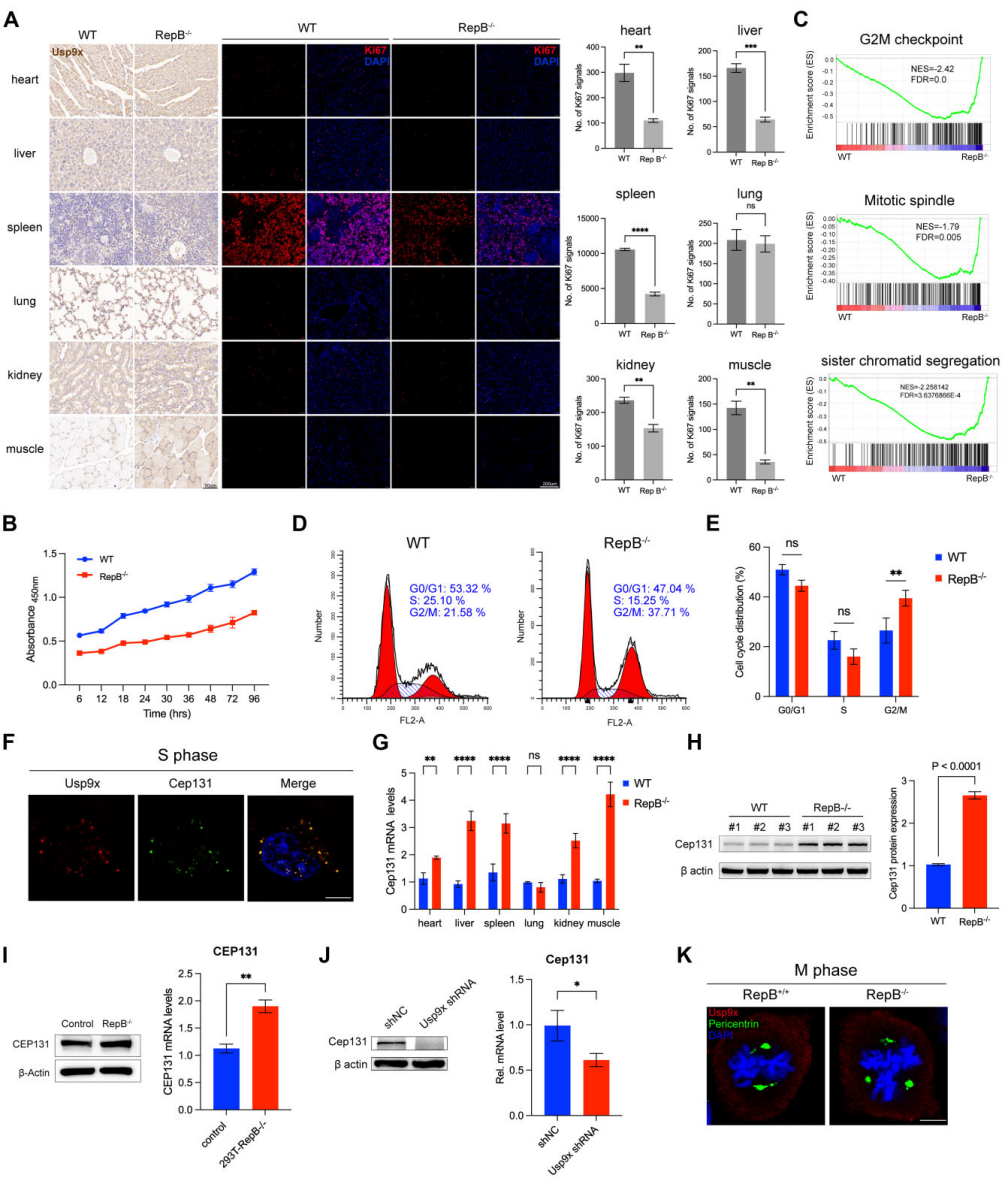
Reduced cell proliferation and mitotic disturbances occur in RepB^−/−^ mice. (**A**) The left panel presents IHC staining of *Usp9x* in various tissues from WT and RepB^−/−^ mice. Ki67 staining performed on various tissue sections is displayed in the middle panel. The quantification of Ki67 staining from the middle panel is presented in the right panel (*n* = 4). ns, not significant, **P < 0.01, ***P < 0.001, ****P < 0.0001. (**B**) Proliferation assessment using the CCK8 assay was conducted on MEFs from WT and RepB^−/−^ mice (*n* = 6). (**C**) Gene set enrichment analysis (GSEA) shows increased expression of cell cycle signature genes in RepB^−/−^ MEFs in comparison to WT MEFs. Normalized enrichment scores and false discovery rates (FDRs) are as indicated and FDR < 0.05 is defined as significant. (**D**) Cell cycle analysis by flow cytometry in MEFs from RepB^−/−^ and WT mice. (**E**) Quantitative analysis of cell cycle phases proportion. ns, not significant, **P < 0.01. (**F**) MEFs were synchronized by double-thymidine block and then released to S phase followed by immunostaining with antibodies against *Usp9x* and *Cep131*. Scale bar, 10 μm. (**G**) *Cep131* mRNA levels across various tissues in WT and RepB^−/−^ mice. ns, not significant, **P < 0.01, ****P < 0.0001. (**H**) *Cep131* protein expression levels in the tails of WT and RepB^−/−^ mice. (**I**) *Cep131* mRNA and protein expression levels in WT and RepB^−/−^ HEK293T cells. **P < 0.01. (**J**) *Cep131* mRNA and protein expression levels in WT and *Usp9x* RNA interference (RNAi) MEFs. *P < 0.05. (**K**) MEFs were synchronized by double-thymidine block and then released to M phase followed by immunostaining with antibodies against *Usp9x* and pericentrin. Scale bar, 10 μm.

We next performed an integrated analysis of the three sample groups. This analysis unveiled three conserved genes—*Usp9x*, *Zdhhc9*, and *Rbm41*—that exhibit upregulated expression following the deletion of repeat B (Fig. 4F and G). Of particular interest, these genes were found to be part of cluster 1, where both H3K27me3 and H2AK119ub marks were completely absent at the loci (Fig. 4H). Considering *Usp9x*’s pivotal function in embryonic development, subsequent gene expression analyses verified its upregulation in multiple tissues from RepB^−/−^ mice, including heart, liver, spleen, kidney, and muscle (Fig. 4I). Protein level assessments further strengthened this finding, demonstrating elevated *Usp9x* protein levels in the tails of RepB^−/−^ mice (Fig. 4J). Immunohistochemical (IHC) analyses further verified *Usp9x* elevation in diverse tissues like heart, liver, spleen, kidney, and muscle (Fig. 5A, left panel). These results underscore the broad impact of repeat B deletion on *Usp9x* expression across various tissue types.

### Deletion of *Xist* repeat B leads to a deceleration of the cell cycle

To elucidate why mice with *Xist* repeat B deletion exhibit reduced body weight and delayed development, we assessed the expression of Ki67, a widely recognized marker of cell proliferation [44, 45]. Immunofluorescence analysis demonstrated a significant decrease in Ki67 fluorescence signals in tissues of mice lacking *Xist* repeat B compared to WT mice, except for the lungs (Fig. 5A), indicating impaired cell proliferation in the affected tissues. To further validate these findings, we employed the Cell Counting Kit-8 (CCK-8) assay to quantitatively assess the proliferation of MEFs *in vitro*. Consistent with the *in vivo* results, MEFs lacking *Xist* repeat B exhibited a significantly reduced proliferation rate compared to WT cells (Fig. 5B). Additionally, GO and KEGG enrichment analyses revealed that differentially expressed genes were predominantly associated with systemic development (Supplementary Fig S6A and B). Furthermore, GSEA analysis identified the enrichment of numerous differentially expressed genes in pathways related to the cell cycle (Fig. 5C and Supplementary Fig. S6C), further supporting the hypothesis that *Xist* repeat B deletion disrupts cell proliferation through modulation of cell cycle-associated pathways.

Therefore, we examined the cell cycle and found that MEFs lacking *Xist* repeat B exhibited an increase in the G2/M phase compared to WT cells (Fig. 5D and E), indicating hindered cell progression in the G2/M phase. Previous *Usp9x* pull-down results showed interaction between *Usp9x* and *Cep131*, a protein involved in centrosome duplication during mitosis [46]. Thus, we synchronized the cell cycle using double thymidine block to detect the interaction between *Usp9x* and *Cep131*. Two hours after thymidine release, immunofluorescence revealed colocalization of *Usp9x* and *Cep131* fluorescence signals (Fig. 5F). Additionally, widespread upregulation of *Cep131* expression was observed in various tissues of mice except for the lungs (Fig. 5G), as confirmed by western blot results (Fig. 5H). Moreover, both RNA and protein levels of *Cep131* were elevated in HEK293T cells lacking *Xist* repeat B (Fig. 5I). Knockdown of *Usp9x* expression in primary MEF cells from mice lacking *Xist* repeat B resulted in decreased levels of both RNA and protein of *Cep131* (Fig. 5J), and mitotic abnormalities were observed (Supplementary Fig. S7A). Additionally, multipolar centrosomes were observed during the metaphase of mitosis in cells from mice lacking *Xist* repeat B (Fig. 5K), which may be one of the key factors contributing to delayed development in RepB^−/−^ mice.

### Deletion of *Xist* repeat B in mice results in altered orientation of ACD

**Figure 6.**
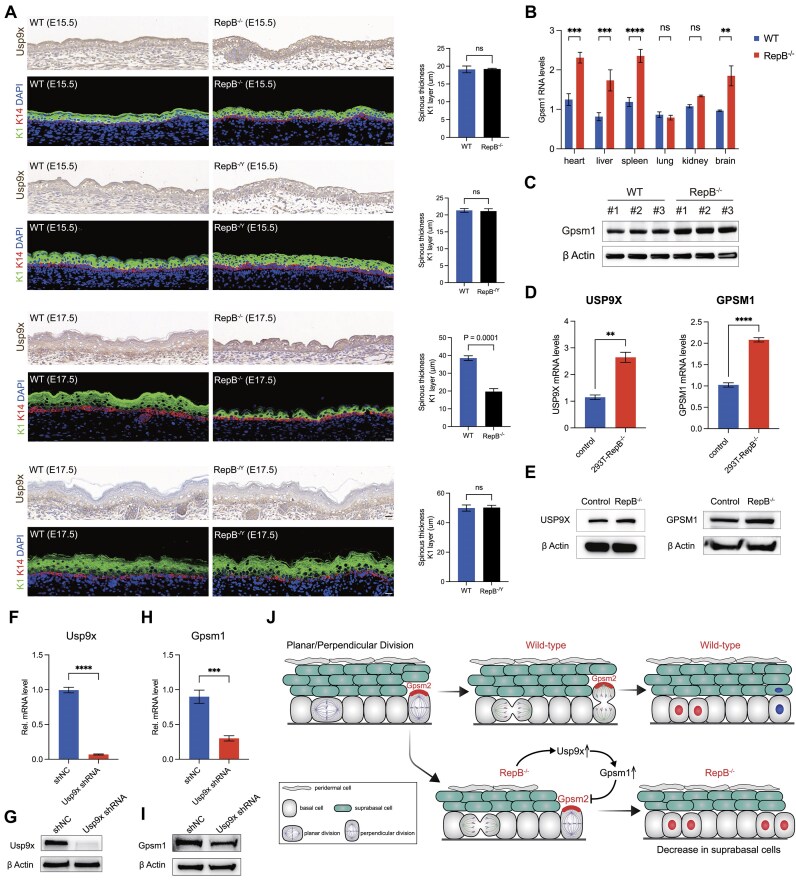
The orientation of ACD is altered in RepB^−/−^ mice. (**A**) IHC staining for *Usp9x* and immunofluorescent imaging of spinous layer thickness (K1, green) in RepB knockout mice of both sexes at E15.5 and E17.5 compared to WT controls (left panel). Quantification of spinous layer thickness (K1, green) of the left panel (*n* = 4, right panel). (**B**) *Gpsm1* mRNA levels across various tissues in WT and RepB^−/−^ mice. ns, not significant, **P < 0.01, ***P < 0.001, ****P < 0.0001. (**C**) *Gpsm1* protein expression levels in the tails of WT and RepB^−/−^ mice. (**D**) *Usp9x* and *Gpsm1* mRNA levels in WT and RepB^−/−^ HEK293T cells. **P < 0.01, ****P < 0.0001. (**E**) *Usp9x* and *Gpsm1* protein expression levels in WT and repeat B knockout HEK293T cells. (**F**) *Usp9x* mRNA levels in WT and *Usp9x* RNAi MEFs. ****P < 0.0001. (**G**) *Usp9x* protein expression levels in WT and *Usp9x* RNAi MEFs. (**H**) *Gpsm1* mRNA levels in WT and *Usp9x* RNAi MEFs. ***P < 0.001. (**I**) *Gpsm1* protein expression levels in WT and *Usp9x* RNAi MEFs. (**J**) Graphical representation of two-step process for oriented cell divisions. Basal progenitors are capable of undergoing either self-renewing planar symmetric divisions (red cell) or differentiative perpendicular asymmetric divisions (blue cell). Polarized apical *Gpsm2* facilitates the formation of apical spindles during metaphase and promotes their perpendicular reorientation during telophase (upper panel). Phenotypes resulting from *Usp9x* and/or *Gpsm1* overexpression in RepB^−/−^ mice (lower panel).

*Usp9x* pull-down results revealed an interaction between *Usp9x* protein and *Gpsm1* (*AGS3*) protein [47]. *Gpsm1* (*AGS3*) is the homologous family protein as *Gpsm2* (*LGN*), which is required for establishing apical polarity during ACD [48–51]. To investigate whether the upregulation of *Usp9x* affects ACD, we examined the skin of mice at E15.5–E17.5 (Basal: K14; supra-basal: K1), a critical period for ACD. The results showed no significant differences between male and female mice lacking *Xist* repeat B compared to WT mice at the initiation stage of ACD (E15.5) (Fig. 6A). However, at the later stage of ACD (E17.5), female mice lacking *Xist* repeat B exhibited a decrease in spinous thickness (decrease in suprabasal cells) compared to WT mice, while male mice showed no significant differences (Fig. 6A). Additionally, IHC results showed significantly higher expression of *Usp9x* in female mice lacking *Xist* repeat B. Furthermore, we detected elevated expression of *Gpsm1* (*AGS3*) in various organs and tissues of mice lacking *Xist* repeat B, except for the lungs and kidneys (Fig. 6B), with WB results also indicating increased expression of *Gpsm1 (AGS3*) (Fig. 6C). In HEK293T cells lacking *Xist* repeat B, mRNA expression of both *Usp9x* and *GPSM1* (*AGS3*) was significantly upregulated (Fig. 6D), and protein expression levels were also elevated (Fig. 6E). Moreover, after knockdown of *Usp9x* expression using shRNA in MEF cells, mRNA and protein expression levels of *Gpsm1* (*AGS3*) were significantly reduced (Fig. 6F–I). Thus, our data demonstrate both *in vivo* and *in vitro* that in female mice, the repeat B of *Xist* is highly correlated with the expression of *Usp9x* and *Gpsm1*.

Therefore, in normal ACD, ACD of basal cells requires *Gpsm2* (*LGN*) to establish apical polarity, resulting in the division of one basal cell and one suprabasal cell. In mice lacking *Xist* repeat B, upregulation of *Usp9x* leads to increased expression of *Gpsm1* (AGS3), which antagonizes *Gpsm2* (*LGN*). *Gpsm1* (*AGS3*) antagonizes *Gpsm2* (*LGN*) in the cortex throughout mitosis, inhibiting perpendicular division, thereby altering the orientation of ACD, resulting in a reduction of suprabasal cells and a decrease in spinous thickness (Fig. 6J). Additionally, we also examined the skin of adult mice, revealing irregular arrangement of spinous layer cells, whereas the spinous layer in WT mouse skin showed a compact and regular arrangement (Supplementary Fig. S7B), indicating altered skin development progression in mice lacking *Xist* repeat B.

## Discussion

The role of *Xist* RNA in orchestrating X-chromosome inactivation (XCI) is well-established, with its distinct domains playing pivotal roles in recruiting chromatin modifiers. In this study, we generated mice lacking *Xist* repeat B, a domain critical for the recruitment of Polycomb group proteins. Interestingly, female mice deficient in repeat B were viable but exhibited a lifelong dwarfism phenotype, contrasting with WT mice. Our data reveal that the deletion of repeat B results in a loss of H3K27me3 and a dramatic reduction of H2AK119ub repressive histone modifications across the X chromosome, highlighting the critical role of *Xist* repeat B in XCI. We then analyzed gene expression changes by binning fold change values in increments of 0.2. A substantial right shift in X-gene expression was also evident. 9.7% (MEFs 91/932), 7.8% (brain 73/932), and 6.6% (liver 62/932) of expressed X-linked genes were up-regulated by ≥1.5-fold. ChIP-seq analysis results showed that reactivation appeared to be context-dependent, with specific loci, such as *Usp9x*, identified as reactivated and potentially contributing to the observed phenotype in repeat B-deficient mice.

It has been reported that repeat B facilitates stable XCI by recruiting the PRC complex [5, 23, 43]. Consistent with these findings, we observed a significant loss of H3K27me3 and H2AK119ub marks on the X chromosome in MEFs derived from repeat B-deficient mice. However, this loss did not result in increased chromatin accessibility, indicating that repeat B deletion does not fundamentally alter the highly compact state of the inactive X chromosome within MEFs nuclei. Previous reports suggested that deletion of repeat B in mESCs results in partial dispersion of *Xist* RNA clouds [4]. In contrast, we did not observe significant changes in *Xist* RNA cloud integrity in primary cells derived from repeat B-deficient mice, suggesting that *Xist* dispersion may depend on chromatin structure or differentiation status. In MEFs, which have a stabilized chromatin state during differentiation, *Xist* RNA dispersion may be less evident compared to the higher chromatin plasticity observed in undifferentiated cells such as mESCs.

Our *in vivo* findings demonstrate that the deletion of *Xist* repeat B results in selective derepression of specific chromatin regions on the inactive X chromosome. These gene clusters rely on *Xist*-mediated Polycomb recruitment for silencing, while others are silenced via Polycomb-independent mechanisms. This suggests that XCI involves multiple parallel silencing pathways, with *Xist* acting through distinct mechanisms to regulate different gene clusters. These insights underscore the regional specificity of X-chromosome imprinting mechanisms [52] and enhance our understanding of the intricate regulatory processes governing XCI during embryonic development.

Polycomb repressive complexes (PRC1 and PRC2) are pivotal in mammalian development and XCI, playing distinct yet complementary roles in epigenetic regulation [53–56]. Our study demonstrates that the impact of repeat B deletion on PRC2 recruitment is more pronounced than on PRC1. Previous reports suggest that PRC1 and PRC2 are independently recruited to Xi [56, 57]. Although we observed a reduction in both H3K27me3 and H2AK119ub upon repeat B deletion, the more drastic reduction in H3K27me3 suggests that PRC2 recruitment may be more dependent on repeat B than PRC1. These findings highlight the complex relationship between the two marks and their recruitment mechanisms. Furthermore, our results highlight the role of CGIs as epigenetic ‘stability islands’ that independently recruit Polycomb complexes. Repeat B-deficient mice exhibit retained H3K27me3 and H2AK119ub marks at certain CGIs, consistent with their ability to serve as Polycomb recruitment hubs [58, 59]. These CGIs may serve as backup mechanisms to ensure stable silencing of key loci in the absence of repeat B. They likely represent primary *Xist* target regions that are sufficient for maintaining localized silencing, while the broader dispersal of epigenetic suppression across the chromosome relies on PRC-mediated mechanisms. This redundancy highlights the complexity of epigenetic regulation on Xi, involving both Polycomb-dependent and independent pathways [52]. Taken together, these results suggest that XCI relies on both Polycomb-dependent and independent pathways, with CGIs and *Xist*-mediated mechanisms providing complementary strategies to establish and stabilize gene silencing across the Xi. This intricate network of epigenetic regulation ensures robust and redundant repression, highlighting the adaptive complexity of XCI across different developmental contexts.

RNA-seq analysis of MEF, liver, and brain tissues revealed that most differentially expressed genes exhibited tissue-specific expression. Notably, *Usp9x*, *Zdhhc9*, and *Rbm41* were consistently upregulated across tissues, suggesting a potential role in tissue development mediated by *Xist* repeat B. Among these, *Usp9x* garnered particular attention due to its approximately one-fold dosage increase in female mice, consistent with recent reports [20]. Elevated *Usp9x* expression correlated with mitotic disturbances and altered asymmetric division of basal keratinocytes in female mice. Given the association of *Usp9x* overactivation with tumorigenesis and neurodegenerative diseases [46, 60, 61], we hypothesize that disruption of *Xist*-mediated XCI may increase female susceptibility to certain diseases. In addition, we compared the gene expression changes observed in our model to those seen in a global *Xist* deletion model during the maintenance phase of X-inactivation, induced in the developing mouse brain using Nestin-Cre driver [62]. Notably, we found that the upregulation of X-linked genes in the repeat B deletion model was more pronounced than in the global *Xist* deletion model (Supplementary Fig. S8A). We hypothesize that this difference arises from the distinct knockout strategies employed in our study compared to previous reports. In our study, we generated a full-body knockout of *Xist* repeat B, whereas prior studies utilized a conditional knockout approach based on the Cre-loxP system. First, conditional knockout methods may not achieve complete deletion in all cells, leaving residual *Xist* expression in some cells, which could mitigate the extent of X-linked gene changes. Second, the conditional knockout was performed after XCI had already been established, whereas our approach deleted repeat B before the initiation of XCI, likely resulting in a more profound impact on the establishment and maintenance of X-inactivation.

The partial reactivation of X-linked genes observed in repeat B-deficient mice underscores the sensitivity of some loci to *Xist*-mediated silencing. Despite these changes, repeat B-deficient mice remained viable and displayed no overt phenotypic abnormalities beyond reduced body size. We propose that *Usp9x* reactivation, leading to cell cycle perturbations and altered cell division, is a key contributor to the dwarf phenotype observed in these mice. Additionally, spindle or centromere dysfunction, linked to chromosomal segregation errors, may elevate tumorigenesis risk, particularly in a sex-specific manner [63–66]. The developmental consequences of other reactivated genes remain to be elucidated and warrant further investigation.

In conclusion, our study demonstrates that deletion of *Xist* repeat B results in widespread loss of H3K27me3 and H2AK119ub modifications and transcriptional reactivation of X-linked genes on Xi, highlighting the critical role of repeat B in maintaining Xi heterochromatin. Given the established links between sex differences and disease susceptibility [18, 20, 21, 67], identifying X-linked genes affected by histone modification changes in repeat B-deficient mice may provide valuable insights for understanding and treating XCI-associated diseases.

## Supplementary Material

gkaf142_Supplemental_Files

## Data Availability

All sequence data can be accessed at the NCBI Gene Expression Omnibus (GEO) using the accession number (GSE278325; GSE278344).

## Supplementary data

Supplementary data is available at NAR online.
