# Supplementary material for: Deletion of Xist repeat B disrupts cell cycle and asymmetric cell division through Usp9x hyperactivation in mice
Source: Nucleic Acids Res. 2025 Mar 5;53(5):gkaf142. doi: 10.1093/nar/gkaf142 (PMC11880805; doi:10.1093/nar/gkaf142)
Supplement: gkaf142_Supplemental_Files [file gkaf142_supplemental_files.zip › Supplementary material.pdf]

Supplementary material

Deletion of *Xist* repeat B disrupts cell cycle and asymmetric cell division through *Usp9x* hyperactivation in mice

Mingming Liang[1,2,†], Lichao Zhang[3,†], Heng Gong[3], Li Yang[3], Haijun Wang[5], Na Song[5], Liangxue Lai[2,3*], Wanhua Xie[4*] and Zhanjun Li[1*]

[1] Laboratory of Organ Regeneration and Transplantation of The Ministry of Education, China-Singapore Belt and Road Joint Laboratory on Liver Disease Research, The First Hospital of Jilin University, Changchun, Jilin, China

[2] CAS Key Laboratory of Regenerative Biology, Guangdong Provincial Key Laboratory of Stem Cell and Regenerative Medicine, Guangzhou Institutes of Biomedicine and Health, Chinese Academy of Sciences, Guangzhou 510530, China

[3] State Key Laboratory for Diagnosis and Treatment of Severe Zoonotic Infectious Diseases, Key Laboratory for Zoonosis Research of the Ministry of Education, Institute of Zoonosis, and College of Veterinary Medicine, Jilin University, 130062, Changchun, China Key

[4] Center for Medical Epigenetics, School of Basic Medical Sciences, Chongqing Medical University, Chongqing 400016, China

[5] School of Basic Medical Sciences, Xinxiang Medical University, Xinxiang 453000, China.

*Correspondence: Zhanjun Li (lizj_1998@jlu.edu.cn), Wanhua Xie (wanhuaxie_18@cqmu.edu.cn), Liangxue Lai (lai_liangxue@gibh.ac.cn)

[†]The first two authors should be regarded as Joint First Authors.

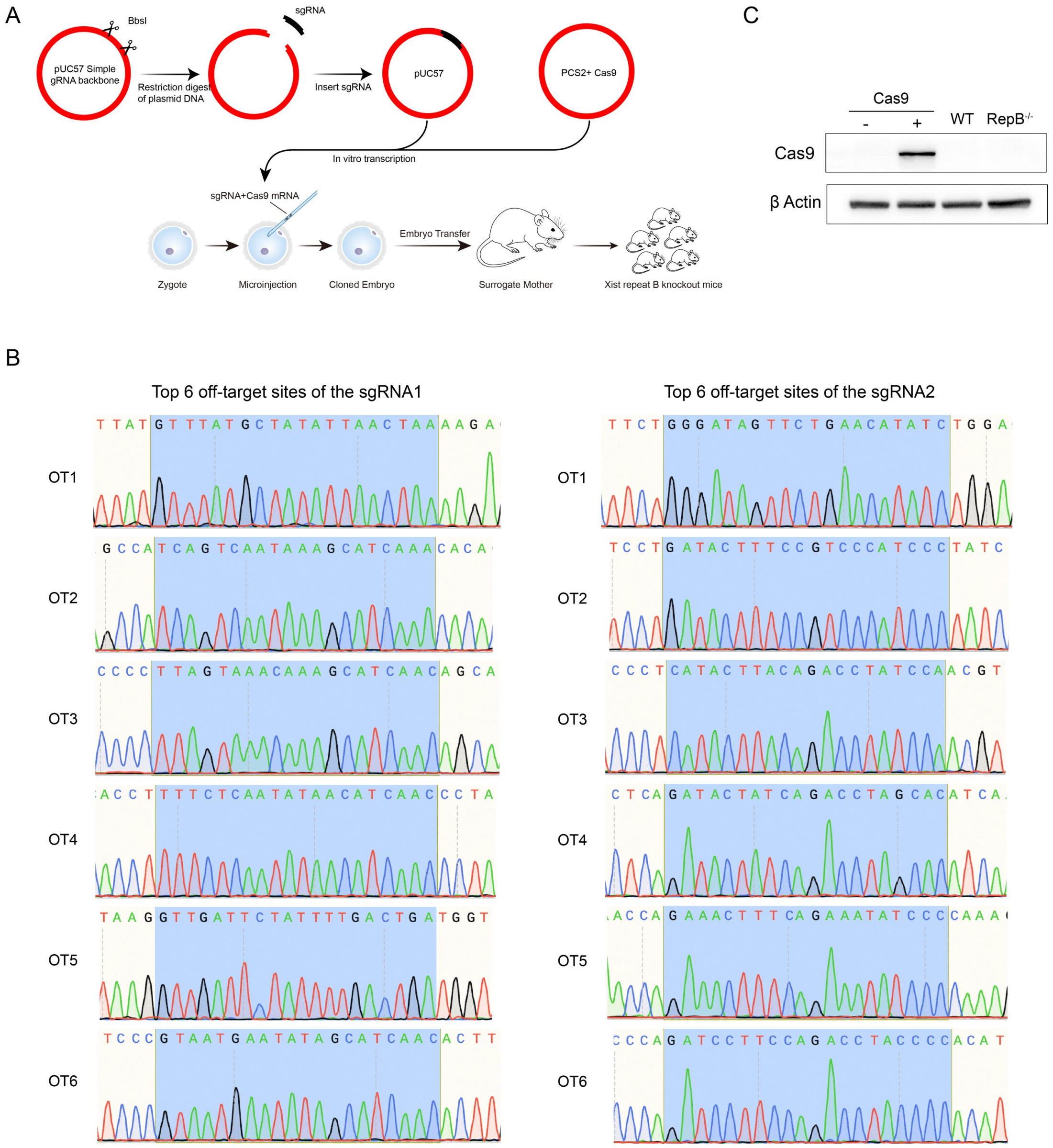

Figure S1. Generation and off-target analysis of the *Xist* repeat B knockout mice.

(A) Schematic of gene editing mouse model generation through microinjection technique.

(B) Off-target sites detection in repeat B knockout mice.

(C) Western blot analysis of Cas9 expression in repeat B knockout mice.

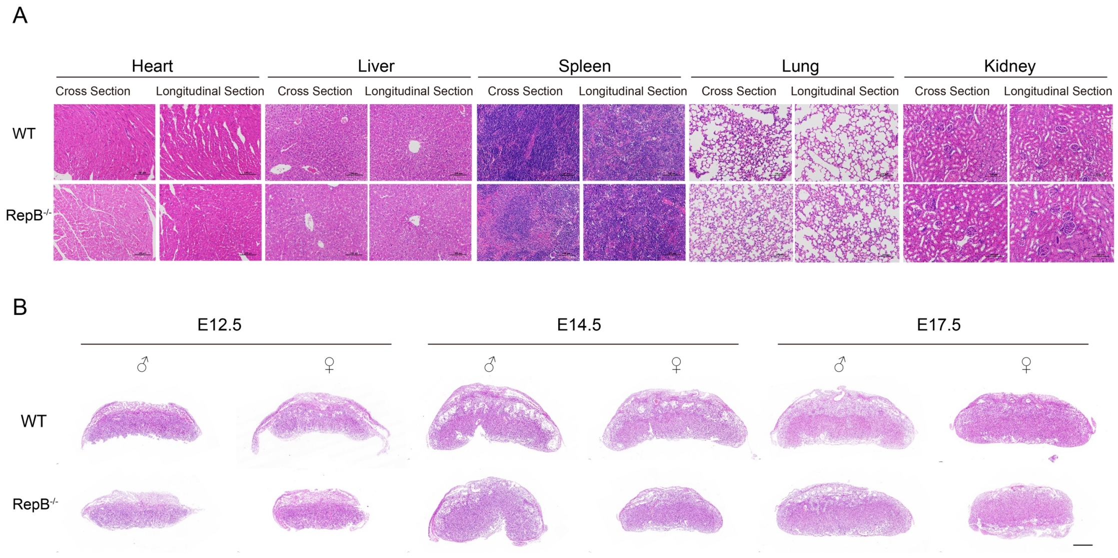

Figure S2. Representative images of H&E staining from WT and RepB$^{-/-}$ mice.

(A) H&E staining of organ sections from mice with indicated genotypes. Scale bar, 100μm.

(B) H&E staining of E12.5, E14.5 and E17.5 placental cross-sections with indicated genotypes. Scale bar, 1mm.

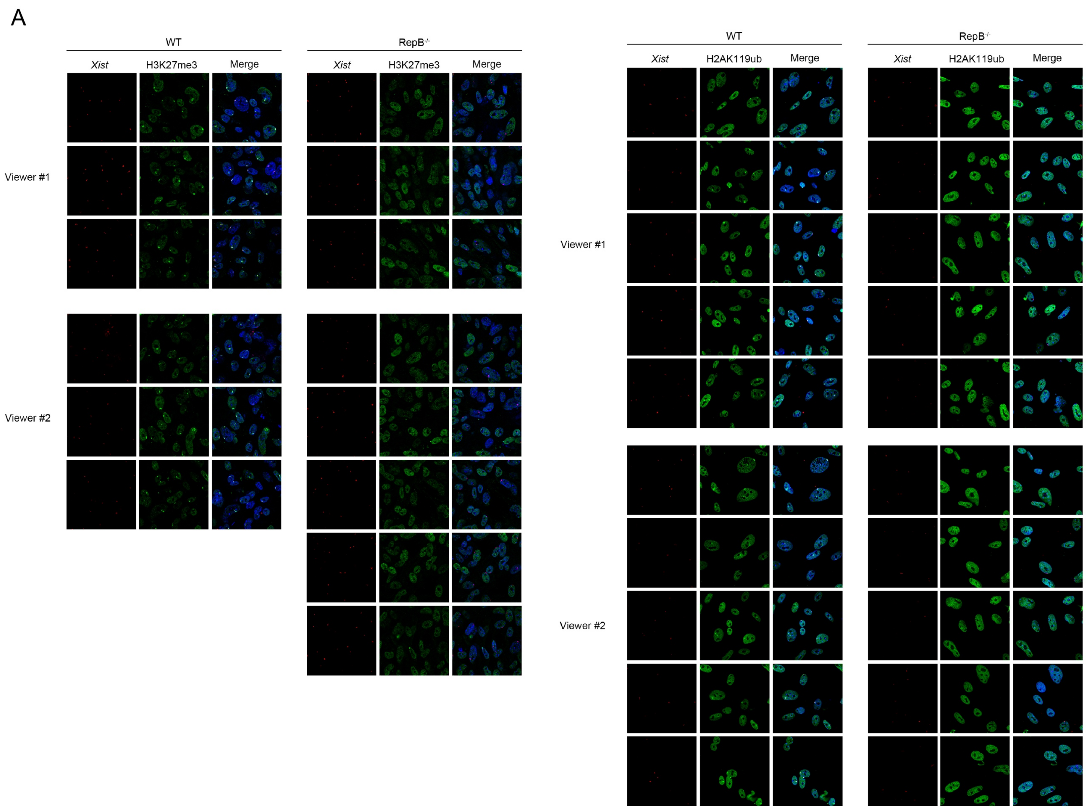

Figure S3. H3K27me3/H2AK119ub IF and *Xist* RNA FISH in RepB$^{-/-}$ and WT MEF cells.

(A) ImmunoFISH analysis of H3K27me3 and H2AK119ub deposition on *Xist* signals using a blinded approach.

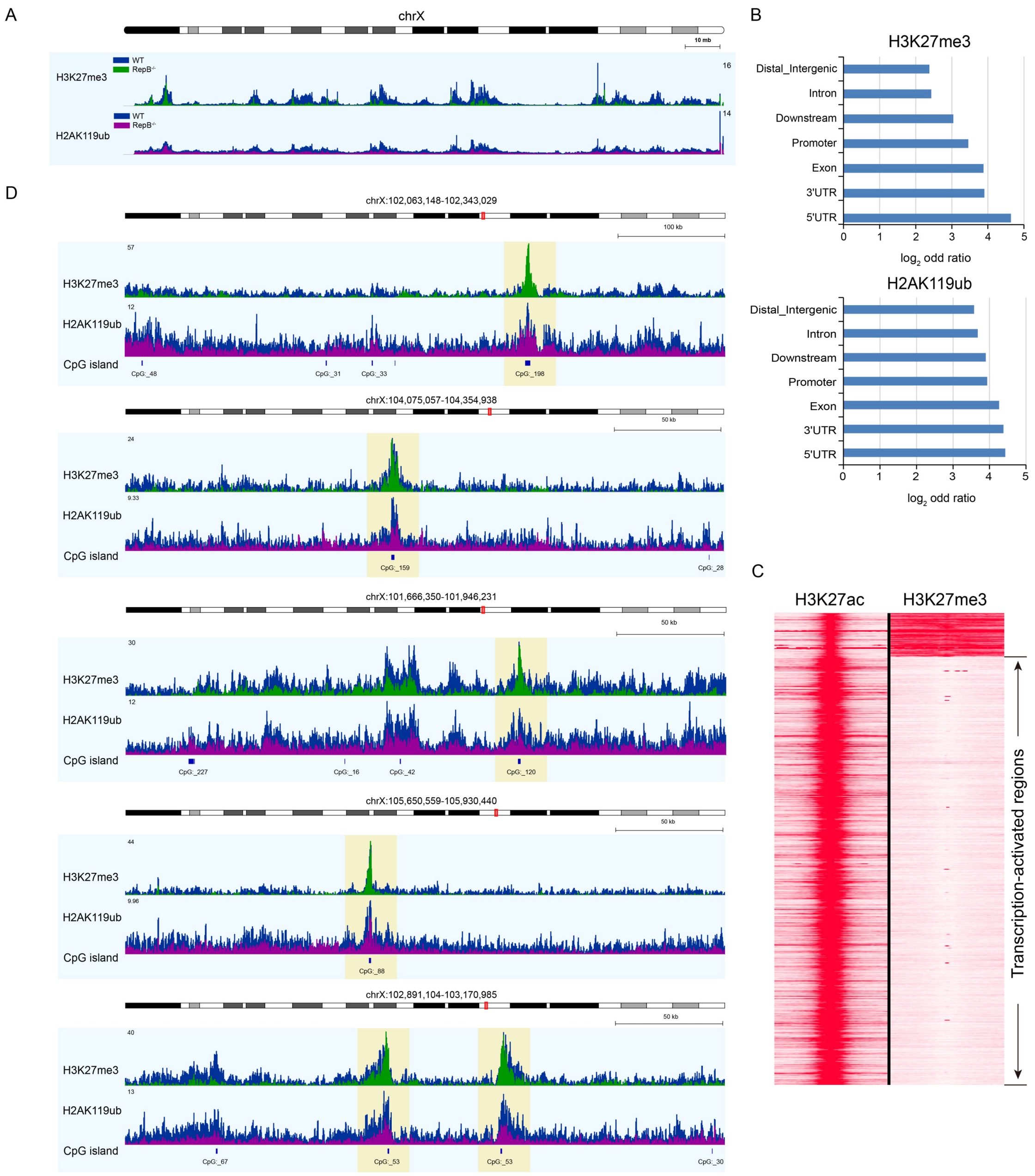

Figure S4. Genome browser tracks showing H3K27me3 (green) and H2AK119ub (purple) accumulation at X chromosome in RepB$^{-/-}$ mice.

(A) Zoom in H3K27me3 (green) and H2AK119ub (purple) accumulation across the chrX regions.

(B) Distribution of down-regulated H3K27me3 and H2AK119ub modifications across intragenic regions in RepB$^{-/-}$ mice.

(C) Screening genome-wide transcription-activated regions for calculating H3K27me3 and H2AK119ub enrichment at transcription-activated regions.

(D) Genome browser view of H3K27me3 and H2AK119ub enrichment at CpG islands in $RepB^{-/-}$ mice.

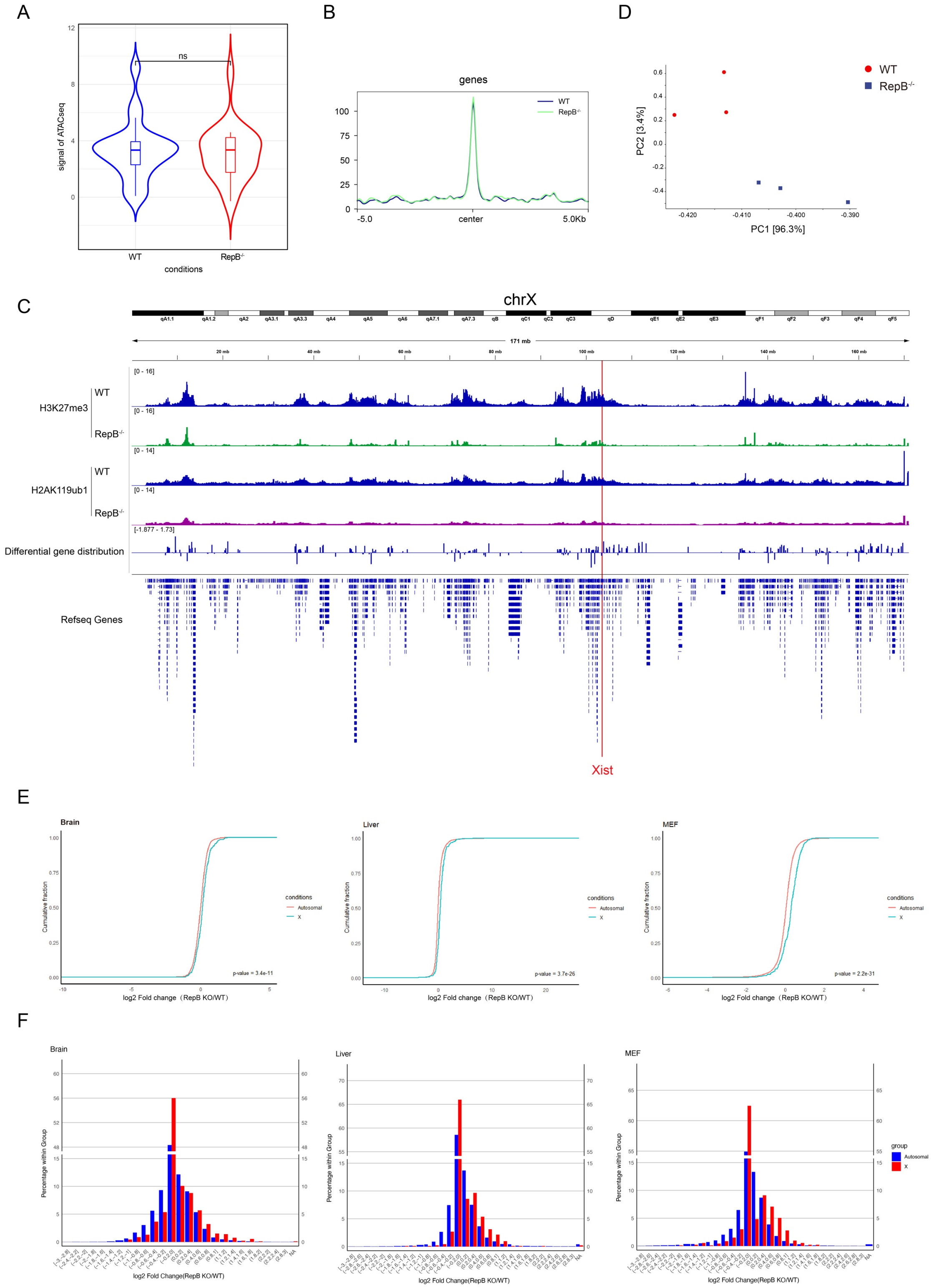

Figure S5. ATAC-seq and differential gene expression analysis on the X chromosome in RepB$^{-/-}$ mice.

(A) Violin plot combined with boxplot showing X chromosome accessibility in RepB$^{-/-}$ mice compared to WT mice. ns, not significant.

(B) Metaplot describing the abundance around summits of ATAC-seq that overlap with genes.

(C) Differential gene distribution of RNA seq on X chromosome in $RepB^{-/-}$ mice compared to WT mice.

(D) Principal component analysis (PCA) plot of RNA seq in MEFs.

(E) Cumulative distribution plots for fold changes in X-linked (green) and autosomal (red) genes in brain, liver and MEF cells.

(F) Distribution of X-linked (red) versus autosomal (black) fold changes in $RepB^{-/-}$ cells relative to control cells. Fold changes are binned in steps of 0.2 (i.e., label of 0.2 on the X-axis includes all genes with fold changes between 0.0 and 0.2). The total protein-coding genes were considered; however, genes with average normalized counts less than 40 in both the wildtype and repeat B deleted groups were regarded as not expressed, with the log2 fold change set to zero.

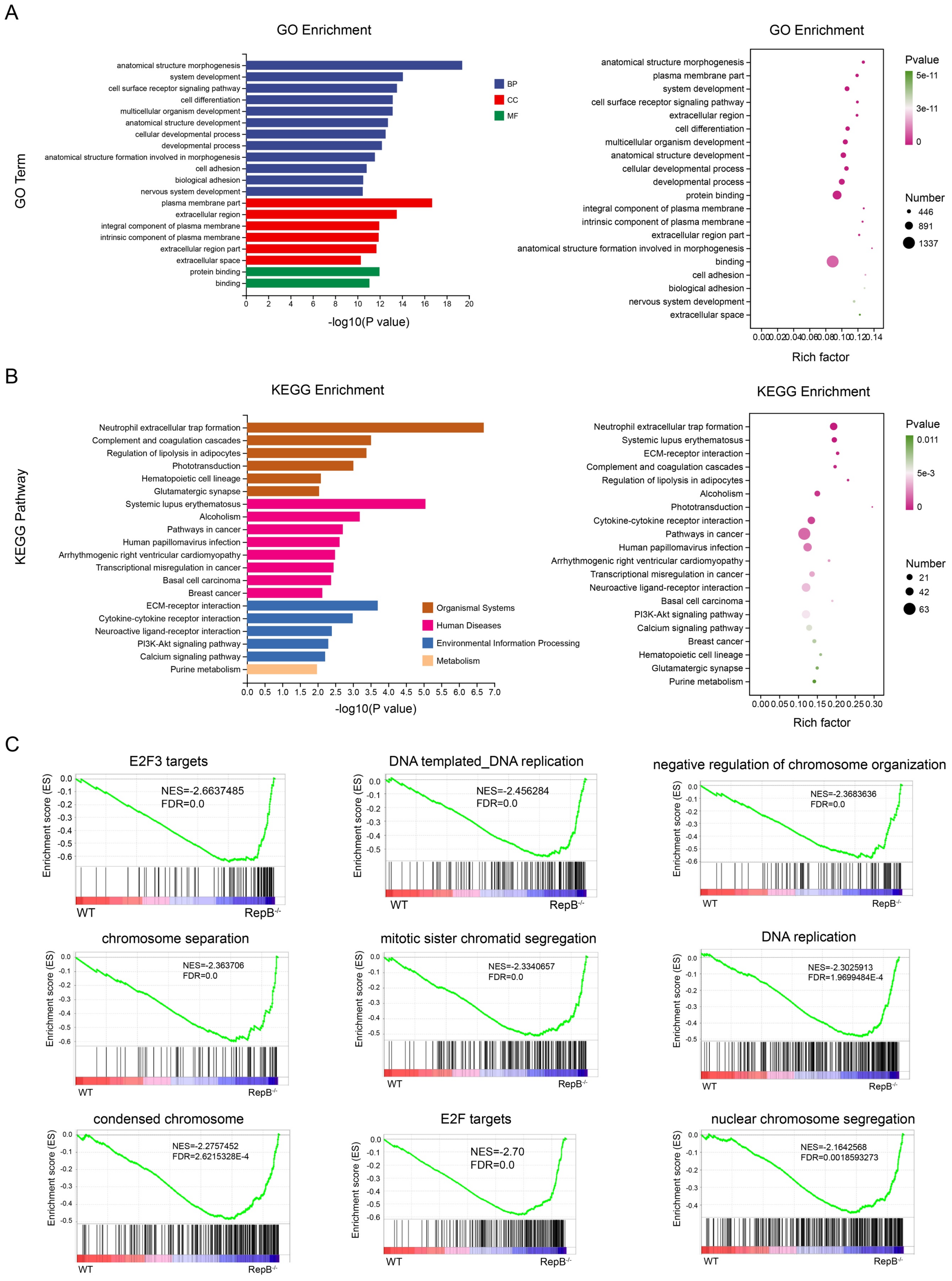

Figure S6. Gene Ontology (GO) and Gene set enrichment analysis (GSEA) of the differentially expressed genes (DEGs) in RepB$^{-/-}$ mice.

(A) GO functional analysis of DEGs.

(B) KEGG pathway analysis of DEGs.

(C) GSEA analysis of cell cycle-related.

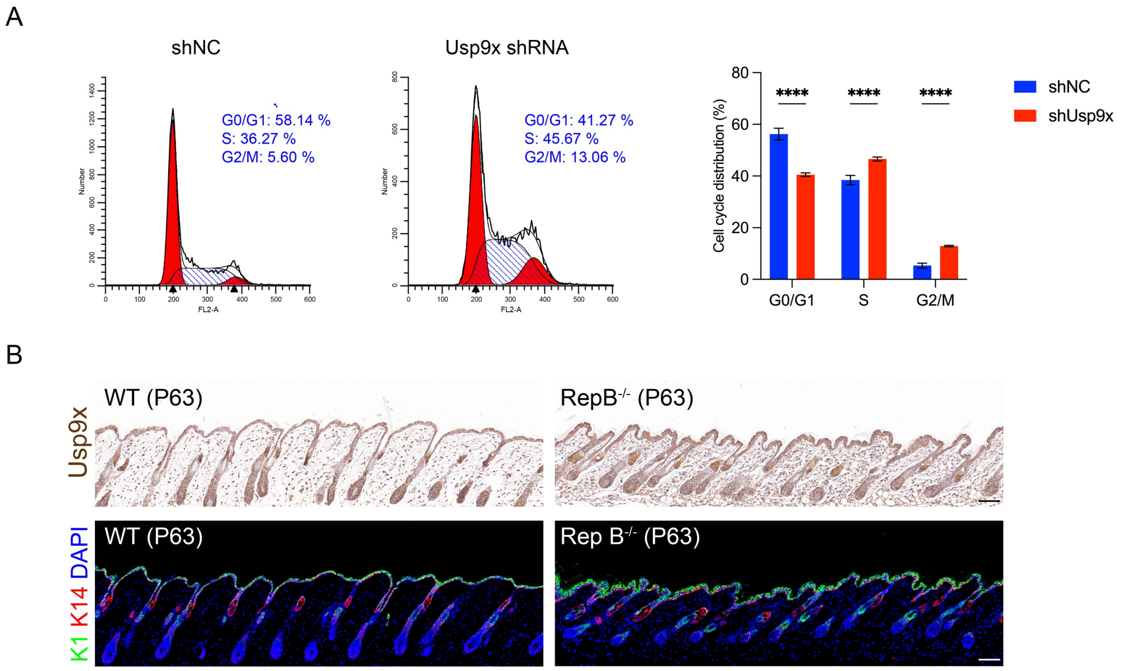

Figure S7. Cell cycle analysis and immunofluorescent imaging.

(A) Cell cycle analysis by flow cytometry in control and *Usp9x* RNA interference (RNAi) MEFs. ****P < 0.0001.

(B) Immunohistochemical (IHC) staining for *Usp9x* and immunofluorescent imaging of spinous layer thickness (K1, green) in RepB$^{-/-}$ mice at P63 compared to WT controls.

A

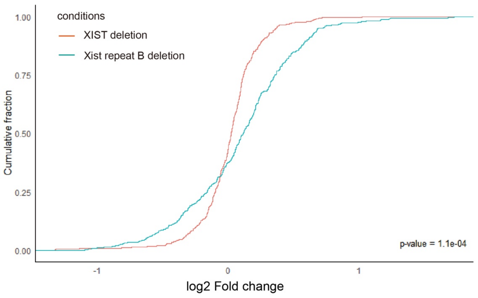

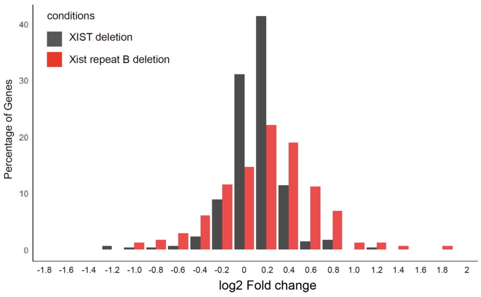

Figure S8. Comparative analysis of X-linked gene expression in the brains of RepB$^{-/-}$ mice and a global *Xist* deletion model using Nestin-Cre.

(A) Cumulative distribution plots for fold changes in X-linked genes (left panel). Fold changes are binned in steps of 0.2 (right panel).
